# Comparison of human chordoma cell-kill for 290 MeV/n carbon ions versus 70 MeV protons *in vitro*

**DOI:** 10.1186/1748-717X-8-91

**Published:** 2013-04-15

**Authors:** Hiroshi Fujisawa, Paula C Genik, Hisashi Kitamura, Akira Fujimori, Mitsuru Uesaka, Takamitsu A Kato

**Affiliations:** 1 Department of Bioengineering, Graduate School of Engineering, The University of Tokyo, 7-3-1 Hongo, Bunkyo, Tokyo, 113-8656, Japan; 2 Department of Environmental & Radiological Health Sciences, Colorado State University, 1618 Campus Delivery, Fort Collins, CO, 80523, USA; 3 Research Development and Support Center, National Institute of Radiological Sciences, 4-9-1 Anagawa, Inage, Chiba, 263-8555, Japan; 4 Research Center for Charged Particle Therapy, Molecular Target Research Unit, International Open Laboratory, National Institute of Radiological Sciences, 4-9-1 Anagawa, Inage, Chiba, 263-8555, Japan

**Keywords:** Particle radiotherapy, Chordoma, OptiCell™, High LET, Carbon, Proton, Bragg peak

## Abstract

**Background:**

While the pace of commissioning of new charged particle radiation therapy facilities is accelerating worldwide, biological data pertaining to chordomas, theoretically and clinically optimally suited targets for particle radiotherapy, are still lacking. In spite of the numerous clinical reports of successful treatment of these malignancies with this modality, the characterization of this malignancy remains hampered by its characteristic slow cell growth, particularly *in vitro*.

**Methods:**

Cellular lethality of U-CH1-N cells in response to different qualities of radiation was compared with immediate plating after radiation or as previously reported using the multilayered OptiCell™ system. The OptiCell™ system was used to evaluate cellular lethality over a broad dose-depth deposition range of particle radiation to anatomically mimic the clinical setting. Cells were irradiated with either 290 MeV/n accelerated carbon ions or 70 MeV accelerated protons and photons and evaluated through colony formation assays at a single position or at each depth, depending on the system.

**Results:**

There was a cell killing of approximately 20–40% for all radiation qualities in the OptiCell™ system in which chordoma cells are herein described as more radiation sensitive than regular colony formation assay. The relative biological effectiveness values were, however, similar in both *in vitro* systems for any given radiation quality. Relative biological effectiveness values of proton was 0.89, of 13–20 keV/μm carbon ions was 0.85, of 20–30 keV/μm carbon ions was 1.27, and >30 keV/μm carbon ions was 1.69. Carbon-ions killed cells depending on both the dose and the LET, while protons depended on the dose alone in the condition of our study. This is the first report and characterization of a direct comparison between the effects of charged particle carbon ions versus protons for a chordoma cell line *in vitro.* Our results support a potentially superior therapeutic value of carbon particle irradiation in chordoma patients.

**Conclusion:**

Carbon ion therapy may have an advantage for chordoma radiotherapy because of higher cell-killing effect with high LET doses from biological observation in this study.

## Introduction

In cancer therapy, the main treatment modalities are, or include, surgery, chemotherapy and radiation therapy. Radiation therapy has been evolving since X-rays were discovered. More recently, intensifying efforts have been invested in devising improved and novel radiation therapy treatments based on innovative technologies and devices [1]. While intensity modulated radiation therapy (IMRT) and image guided radiation therapy (IGRT) using photons are two outstanding examples of such recent developments in radiation therapy [1], the most notable instance in the field of a vastly improved therapeutic gain would be that derived from charged particle radiation therapies with accelerated carbon ions or protons [1]. Accelerated carbon ions and protons have been very successfully used for treating solid cancers primarily due to their advantageous property of excellent physical dose-distribution and deposition around the Bragg peak [2,3]. Several recent clinical studies have reported that outcomes from charged particle beam radiation therapy yield equivalent or superior outcomes to surgical modalities in a number of cases such as prostate, lung, head and neck, bone and soft tissue tumors [4,5]. In bone tumors, namely chordomas, malignancies appear to be highly responsive to charged particle radiation therapy which also maximally preserves adjacent organs and their function, whereas the latter are difficult to preserve through unavoidably highly invasive surgery [5-9].

Chordomas are primary malignant bone tumors that arise from vestigial notochordal remnants and appear more commonly in the clivus, the basilar process of the occipital bone and the sacrum or coccyx, vertebrae at the caudal region of the spine [10,11]. Chordomas represent only 1 to 4% of all primary malignant tumors and among their hallmark characteristics are containment and slower growth kinetics together with less frequent metastases [12]. While the first-line therapy of choice for chordoma treatment remains surgery, radiation therapy is also used [4,13,14] because of the deeply ensconced anatomical location of these tumors and the severe negative quality of life issues resulting from surgical resections [6]. Radiation therapy, especially in the case of charged particle beams involving carbon ions or protons, has been commanding much attention of late particularly in the treatment of bone and soft tissue sarcomas [6,15]. Both carbon ions and protons have thus been successfully used in the treatment of chordomas, and their superior therapeutic outcomes have been reported in clinical practice [4,8,15]. Notwithstanding these remarkable advances with charged particle beam therapies of chordoma, there remains a severe paucity of basic biological data on this tumor cell type. Basic scientific investigation into the biology of these tumors has been hampered due to several limiting characteristics of their primary cell type, namely their long mean doubling time and the lack of types corresponding cell lines [16,17]. Nonetheless, a few noteworthy cytogenetic studies describing the karyotypes and chromosomal aberrations through multicolor fluorescence *in situ* hybridization and array have been reported [18,19]. We also have previously reported that U-CH1-N, a newly established chordoma cell subline from U-CH1, is very sensitive to high Linear Energy Transfer (LET) radiation involving charged particle beams such as those of carbon, neon, silicon, argon and iron ions [16]. To date, however, the sensitivity of chordoma tumors to carbon and protons remains ill-defined.

In this study, we compare the relative biological effectiveness (RBE) of carbon ion particles to that of proton particles in a human chordoma cell line, UCH1-N, cultured in the OptiCell™ system. We previously established and characterized this cell line *de novo*[16] with a notably abridged doubling time of approximately 3 days compared to that of the original 7 days in the originating cell line. The OptiCell™ system is a commercially available cell culture technology that was designed as a unique cell culture format with respect to growing, monitoring, and transporting of cells in culture [20]. These vessels consist of an enclosed sterile cell culture chamber with two parallel gas-permeable cell culture-treated polystyrene membranes. The thin profiles of these chambers (2 mm in width) offer very specific advantages. More detailed acquisition of biological data at each position (LET and dose) is possible with respect to experimental particle beam pathways.

The present study is the first to undertake and report a direct comparison between the cell killing effects of 290 MeV/n carbon ions and 70 MeV protons in a chordoma cell line in a clinically relevant *in vitro* system. Our data lead to a description of chordomas as an even more radiation resistant solid tumor than previously understood, which responds significantly to carbon particle beams in an LET and dose dependent manner rather than to protons, with an enhanced RBE greater than one. Further work will be needed to determine whether this characterization applies to other tumor types and beam qualities and doses. Finally, our data suggest that estimates of cell killing in a classical cell culture system overestimate the responsiveness of tumor cells to all radiation qualities, particularly in the case of protons and strongly support the heightened relevance of carbon beam particle therapy as the optimal treatment of chordoma tumors in the clinic.

## Methods and materials

### Cells and culture

The human chordoma cell line U-CH1 was kindly supplied by the Chordoma Foundation (Greensboro, NC, USA). The U-CH1-N cells used in this study are a subpopulation derived from the original U-CH1 chordoma cells at National Institute of Radiological Sciences (NIRS) [16]. Cells were grown in α-MEM (Invitrogen, Carlsbad, CA, USA) supplemented with 10% (v/v) fetal bovine serum (Sigma, Japan) and 1% (v/v) antibiotics and antimycotic (Invitrogen, Carlsbad, CA, USA) and they were maintained in a tissue culture incubator at 37°C in a 100% humidified atmosphere of 5% CO_2_ in air.

### Irradiations

**Figure 1:**
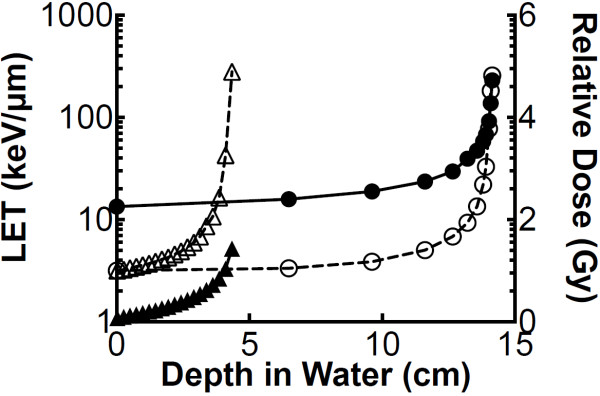
**Dose and LET at each depth in water.** Open circles represent relative dose of proton (70 MeV) compared to entrance, and closed circles represent LET spectrum of proton. Open triangles represent relative dose of carbon ions (290 MeV/n) compared to entrance, and triangles represent LET spectrum of carbon ions.

Hadron irradiations were conducted at the NIRS in Chiba, Japan. Carbon ions were accelerated to 290 MeV/n using the Heavy Ion Medical Accelerator in Chiba (HIMAC) and protons were accelerated to 70 MeV using the NIRS-930 cyclotron delivery port in C-8. Dose rates for carbon ions and protons were set at 1 Gy/min. Monoenergetic 290 MeV/n carbon ions have a LET value of 13 keV/μm on entrance. Monoenergetic 70 MeV protons have a LET value of 1 keV/μm on entrance. Maximum doses of carbon ions and protons were delivered at depths of 14 and 4 cm in water, respectively (Figure 1) [21]. X-ray irradiations were performed using a TITAN irradiator at 200 kVp, 20 mA and 0.5 cm aluminum and copper filters (Shimadzu, Japan), at a dose rate of 1 Gy/min. Gamma-ray irradiations were carried out at a dose rate of 2.5 Gy/min at Colorado State University (Fort Collins, CO, USA) using a 6,000 Ci (nominal activity) ^137^Cesium sealed source [Model Mark I-68A (SS0056) J.L. Shepherd, San Fernando, CA, USA]. Irradiations were carried out at room temperature.

### Cell survival assays using OptiCell™ culture chambers

Cultured cells were trypsinized and re-suspended into complete growth medium. Once re-suspended, 10 ml of medium containing 8,000 cells was placed into each individual OptiCell™ cell culture vessel (Thermo Fisher Scientific, Pittsburgh, PA, USA) within 3 hours prior to irradiation. The chamber dimensions were 2 × 65 × 150 mm with a maximum 10 ml volume capacity. All samples were stacked in layers and arranged perpendicular to the beam paths. Immediately following radiation at doses of 1, 2 and 3 Gy (of exposure for the first chamber) with carbon and proton particle beams and of 2, 4, 6 and 8 Gy with gamma-rays, cells were incubated at 37°C in 100% humidified atmosphere of 5% CO_2_ in air for 3 weeks. After this culture period, cells were washed with 0.9% NaCl, fixed in 100% ethanol and stained with 0.1% crystal violet. Colonies containing more than 50 cells were recorded as reproductively viable surviving cells. Cell survival assays were carried out in as many as four independent replicates.

### Cell survival assays using standard culture dishes

Exponentially growing U-CH1-N cells cultured in T12.5 flasks (Becton Dickinson, Franklin Lakes, NJ, USA) were irradiated with 70 keV/μm of near Bragg peak carbon ions, entrance region protons or X-rays at room temperature, then trypsinized and plated onto 100 mm cell culture dishes. They were kept in an incubator for three weeks without medium change. Up to 4 replicate experiments were conducted and after colonies were formed, cells were fixed and stained as described above.

### Statistical analysis

Data were analyzed using Prism 5™ (GraphPad, La Jolla, CA, USA). Standard errors of the means for data were calculated and were depicted in each figure.

## Results

### Cell survival versus depth in water

**Figure 2:**
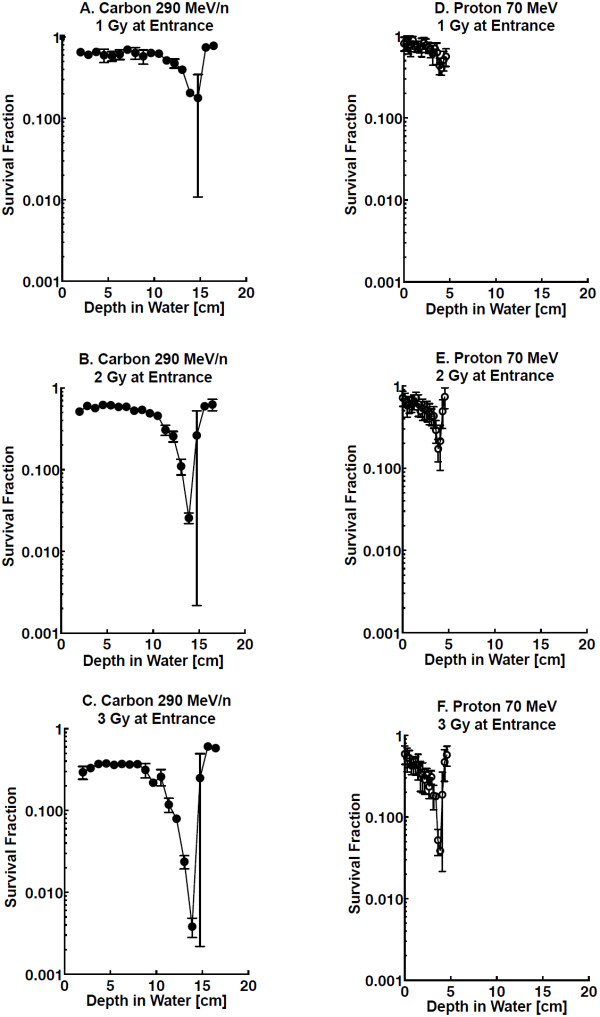
**Cell survival curves at each depth in water.** Cell survival assays were conducted with stacked OptiCell™ cell culture systems, irradiated with 290 MeV/n carbon ions: **A-C** and 70 MeV protons: **D-F**. Error bars indicate standard errors of the means from as many as four independent experiments.

Cell survival assays were conducted with our stacked OptiCell™ cell culture system, and cells irradiated with carbon ion and proton particles. Positions of OptiCell™ chambers at each depth were altered to the depths in water using the physical parameters determined in our previous report [21]. Bragg peaks of each carbon and proton beams were delivered at depths of 14 and 4 cm, respectively (Figure 1). Survival fractions were gradually decreased and minimized at the Bragg peaks. Survival fraction near the Bragg peaks for carbon showed lower survival fractions compared to those for protons (Figure 2A-F).

### Cell survival at low LET and at high LET

**Figure 3:**
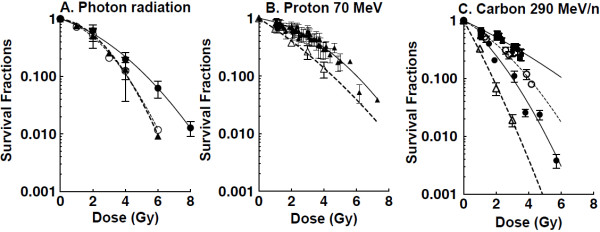
**Dose response curves for proton and carbon ions.** Survival fraction per dose obtained by OptiCell™ was calculated from Figure 1 and 2. Cell survival curves in standard cell culture dishes are also plotted. **A**. photon radiation, gamma-ray survival obtained by OptiCell™ was described by closed circles. Standard survival assay for x-ray was described by closed triangles and gamma-rays was described by open circles. **B**. proton, closed triangles represent OptiCell™ survival and open triangles represent standard survival. **C**. carbon ions, closed squares represent LET 13–20 keV/μm, open circles represent LET 20–30 keV/μm, closed circles represent LET >30 keV/μm obtained by the OptiCell™ system. Open triangles represent monoenergetic carbon ions LET 70 keV/μm. Error bars indicate standard errors of the means from as many as four independent experiments.

Cell survival in the OptiCell™ system for U-CH1-N cells was evaluated in response to three groups of LET values for carbon particles. LET values were assigned to 13–20, 20–30, and >30 keV/μm value ranges. Higher LETs killed more cells. Proton particles were considered to be of low LET value only. Depths in water were translated to radiation doses by referring to physical parameters as our previous report (Figure 1) [21]. The survival fractions that correspond to the doses were then plotted (Figure 3A-C). Cellular lethality caused by carbon ions was found to depend on both the dose and the LET, while in contrast, that caused by protons depended exclusively on the dose in the range of LETs in our study (1 to 10 keV/μm).

### RBE values for U-CH1-N

RBE values were calculated based on the D_10_ values, at doses resulting in one decade of cell killing. RBEs were obtained from D_10_ values for gamma-rays divided by the D_10_ for each ion particle. Carbon ions displayed the highest RBEs in this charged particle study: LET >30 keV/μm 290 MeV/n carbon ions had an RBE of 1.69 and lower LET (13–20 and 20–30 keV/μm) 290 MeV/n carbon ions showed an RBE of 0.86 and 1.27 respectively. The proton’s RBE value was 0.89 and very similar to LET 13–20 keV/μm carbon ions.

### Cell survival and RBE in standard cell culture

Survival fractions following irradiation of U-CH1-N were also obtained using classic cell culture dishes. Both gamma-rays and x-rays were used for the standard photon radiation. U-CH1-N showed very similar sensitivity to both kinds of photon radiation. Survival fractions were shown to depend on radiation dose, decreasing as doses increased (Figure 3). The entrance region of 70 MeV proton cell survival curves showed similar profiles to those for gamma-rays and x-rays, while in contrast, carbon ions survival curves revealed a markedly more sensitive response to carbon ions than to photon radiation. The calculated RBEs based on D_10_ for carbon ions and protons against gamma-rays (D_10_ = 4.11) and x-rays (D_10_ = 4.12) in this second *in vitro* system were higher than those determined in the OptiCell™ system and were of 2.27 and 0.90, respectively.

## Discussion

Chordomas grow relatively slowly *in vitro* and the doubling time of human chordoma U-CH1 cells *in vitro* is reported as 7 days [16]. Because of its characteristic kinetics as slow-growing, radiobiological characterization of chordoma cells is still lacking, though many clinical trials have been conducted and their outcomes reported [6,16]. Recently, however, an established cell line with a shortened doubling time of 3 days was developed with cell cycle kinetics thereby of particular usefulness towards acquiring invaluable biological information on chordoma cell growth and sensitivity to radiation or anti-tumor drugs *in vitro*[16] and ultimately, *in vivo*.

In the present study we chose to focus on the cell survival of chordoma cells *in vitro* irradiated with two very different clinically relevant hadron beams in terms of quality, namely carbon ions and protons, currently used for charged particle radiation therapy in a limited number of highly specialized centers throughout the world [5,22-24]. Qualitatively and quantitatively, our understanding of chordomas and data accrual for carbon treatments of this tumor lag far behind those for protons in light of the much smaller number of carbon treatment facilities worldwide. In this study, we show that exposure to carbon ions results in cell survival fractions lower than those for protons near the Bragg Peak. In the OptiCell™ system, RBE values against gamma-rays were 1.69 for higher LET (>30 keV/μm) carbon ions, 0.85 for low LET (13–20 keV/μm) carbon ions, 1.27 for low LET (20–30 keV/μm) carbon ions, and 0.89 for the entire range of protons (1-10 keV/μm) (Figure 3) and thereby demonstrate a marked superiority of carbon to proton charged particles in killing chordoma cells, explaining at least in part carbon’s superior ability to treat tumors in the clinic. Dose–response curves also show these differences between carbon ions and protons. While the latter *in vitro* cellular determinations reflect the clinical course of these actual chordoma tumors following carbon versus proton therapy *in vivo*, they do define a larger quantitative therapeutic differential between the two particle beams in question.

The cell killing effectiveness of carbon ions in our study reveals for the first time a dependency of the chordoma cell lethality of these hadrons on both the radiation dose and the particles’ LET values in a three-dimensional *in vitro* setting [25]. The present study extends and refines the qualitatively similar effects of carbon ions in a standard tissue culture environment as we have previously shown [16]. A marked novelty and advantage of our irradiating U-CH1-N cells in stacked OptiCell™ chambers with accelerated carbon beams is that we concurrently include both high LET particles with values near the Bragg peak and low LET particles with values outside of the Bragg peak area. In this study, we show that high LET carbon beams which are at present used in clinical treatment [26] do indeed kill more U-CH1-N cells than do low LET carbon particle beams. Moreover, we show that while proton particle beams which are considered to be of low LET also result in a cell-kill profile which consists of both a plateau and a Bragg peak areas, the dose response curve for chordoma cell-kill as a function of exposure to protons presents no significant differences when the survival data are segregated into outside- and near-Bragg peak areas. Thus, low LET protons and carbon are noticeably similar effective in killing chordoma cells (proton D_10_ = 0.89 and 13–20 keV/μm carbon ions D_10_ = 0.85). Cell kill data following proton beam exposure in our study, moreover, suggest that the effect of protons on cell kill would be far less therapeutically controllable or modifiable than that of a carbon beam as the former depends on the dose of alone. From the results above, the use of carbon ions in particle therapy of chordoma offers a distinct advantage with respect to both effectiveness and efficiency in that its therapeutic gain depends on LET and a demonstrated higher cell killing. These tendencies confirm chordoma cells patterns of superior cell kill by carbon ions recently shown in other cell lines, namely Chinese Hamster Ovary (CHO) cells [21] and human salivary gland cancer HSG cells [27].

OptiCell™ tissue culture chambers are cell culture systems optimized for cell growth, culture monitoring, and transportation. They are designed for maximizing tissue culture incubator space in light of their narrow cross-sectional profile and their highly specific enclosed-system which is readily applied to edifying three-dimensional stacks of monolayer culture systems as *in vitro* models for cell [20,28]. In the present study, we devised stacked three-dimensional *in vitro* OptiCel™ arrays in configurations modeling *in vivo* clinically relevant anatomical constructs which we then used to determine cell survival fractions at each depth of beam energy deposition. Thus, development and alignment of these live-cell biological phantoms in three dimensions along a beam path more relevantly mimic a desired intravital geometry. To study relative *in vitro* survivals at varying depths, several other physical culture methods have been used, including Petri dishes fixed in a particular device [29], and a “cell stack chamber” [30]. Relative to these other specialized cell culture methods, the most advantageous feature of the stacked OptiCell™ chamber system is the acquisition of many more - and greater detailed - data points, with an optimal resolution of 2 mm. Since the maximum dose of protons accelerated to 70 MeV was delivered at a depth of ~4 cm in this study, a detailed analysis was necessary to acquire data near the proton Bragg peak. One significant difference between our present study protocol and previously used methods is the relative timing of cell plating and that of irradiations. There are essentially two different approaches to performing studies involving colony formation assays. A first strategy involves the plating cells before exposure to drugs or radiation, while the other relies on treating cells prior to subsequent passaging them to assess their resulting clonogenic ability [31]. Survival fractions are known to depend on whether plating of cells is performed before or after irradiation [32,33]. In the present study, cells were diluted and plated prior to irradiation and maintained undisturbed for the subsequent three weeks, which contrasts with the methodology employed in our previously reported study [16]. In this study, cell survival fractions following carbon or proton irradiation within OptiCell™ chambers demonstrate a marked radioresistance when compared with cells cultured in standard tissue culture dishes (Figure 3). The survival difference in both culturing conditions can also be explained in part by the respective sequences of plating and irradiation.

In summary, this is the first report of a direct and therapeutically relevant *in vitro* comparison between the cell killing effects of carbon ions and protons on human chordoma tumor derived cell lines. Our data describe an experimental setting in which we have begun to investigate the superior therapeutic index of high LET carbon charged particle beams over all other charged particle and photon radiation modalities. We contend that use of the OptiCell™ system provides an overall more reliable understanding of the biology of cells within a native *in vivo* chordoma tumor environment and should form the basis of future chordoma studies.

## Conclusion

Our chordoma model describes a more biologically representative assessment of the true radiation responsiveness or resistance to varying radiation qualities (high versus low LET carbon relative to protons and photons) and doses with respect to charged particle beams at energies and at doses currently used in the clinic. Our data for a rare and difficult to treat chordoma tumor type indeed confirm that chordomas are exquisitely more responsive targets to carbon ion than to proton radiotherapy from an *in vitro* biological standpoint, principally in light of a unique dependency of carbon ion effects on both LET and dose on cellular lethality.

## Competing interests

The authors declare that they have no competing interests.

## Authors’ contributions

HF wrote the manuscript and performed all of the experiments. PCG minimally assisted with experiments and wrote and edited the manuscript. HK aided in the experiments of proton including irradiation and measurement. AF aided in the experiments of carbon at the HIMAC. MU analyzed the experiments and edited the manuscript. TAK conceived and performed the experiments, edited the manuscript and supervised the entire project. All authors discussed the results and commented on the manuscript. All authors read and approved the final manuscript.
